# Relationships Between Physicochemical and Structural Properties of Commercial Vermiculites

**DOI:** 10.3390/ma18040831

**Published:** 2025-02-14

**Authors:** Ayoub Lahchich, Pedro Álvarez-Lloret, Javier F. Reynes, Celia Marcos

**Affiliations:** 1 Departamento de Geología, Facultad de Geología, Universidad de Oviedo, C. Jesús Arias de Velasco s/n, 33005 Oviedo, Spainpedroalvarez@uniovi.es (P.Á.-L.); 2 Departamento de Química Orgánica e Inorgánica, Facultad de Química, Universidad de Oviedo, Av. Julián Clavería, 8, 33006 Oviedo, Spain; fernandezreyjavier@uniovi.es

**Keywords:** vermiculites, heating, microwave irradiation, hydrothermal, mechanochemical

## Abstract

This study examines the effects of thermal (1000 °C), hydrothermal (100 °C), mechanochemical (ambient T), and microwave (~100 °C) treatments on three types of Chinese vermiculites, one with lower potassium content than the others. The goal was to obtain materials with enhanced properties related to specific surface areas. The response of the vermiculites to treatments and their physicochemical properties were analyzed using X-ray diffraction (XRD), thermal analysis (TG and DTG), and textural characterization via the BET method. XRD analyses showed similar mineral composition in treated and untreated samples, but the treatments affected the intensity and width of phase reflections, altering crystallinity and structural order, as well as the proportions of vermiculite, hydrobiotite, and phlogopite. Thermogravimetric analysis revealed two mass loss stages: water desorption (from 25 °C to about 250 °C) and recrystallization or dehydroxylation (above 800 °C). The isotherms indicated mesoporous characteristics, with hydrothermally CO_2_-treated samples having the highest specific surface area and adsorption capacity. The samples with vermiculite, hydrobiotite, and phlogopite generally showed moderate to high specific surface area (S_BET_) values, and mechanochemical treatments significantly increase S_BET_ and pore volume (V_p_) in the vermiculite and hydrobiotite samples. Crystallinity affects S_BET_, average V_p_, and average pore size, and its monitoring is crucial to achieve the desired material characteristics, as higher crystallinity can reduce S_BET_ but improve mechanical strength and thermal stability. This study highlights the influence of different treatments on vermiculite properties, providing valuable insights into their potential applications in various fields (such as thermal insulation in vehicles and aircraft, and the selective adsorption of gases and liquids in industrial processes, improving the strength and durability of building materials like cement and bricks).

## 1. Introduction

Vermiculite is a silicate mineral belonging to the phyllosilicate subclass. It resembles micas in appearance and exhibits a range of colors from green to yellow and brown. It typically exhibits a leafy habit, a Mohs hardness of approximately 2, and a density between 2.4 and 2.7 g/cm^3^. Structurally, it belongs to the 2:1 group [1], consisting of two T-O-T layers connected by an interlayer. The T-O-T layer includes an octahedral sheet of Mg^2+^ situated between two tetrahedral sheets of Si^4+^. The interlayer comprises an octahedral sheet of Mg^2+^ bonded to oxygen or OH^−^ groups and containing water. Isomorphic substitutions, particularly the replacement of Si^4+^ with Al^3+^ in the tetrahedral sheets, are common. Vermiculite can undergo hydration and dehydration processes due to the presence of water and OH^−^ groups, influenced by factors like temperature, pressure, particle size, relative humidity, and chemical composition [1,2,3,4,5,6,7,8,9,10,11,12,13,14]. In addition to pure vermiculite, there are “commercial vermiculites”, which consist of various interstratifications of mica/vermiculite, vermiculite with different states of hydration, mixtures of mica and vermiculite, etc., with a layered structure composed of different ordered or non-ordered phases. These mixtures have a mosaic-like distribution of phases. The key feature of commercial vermiculites is their ability to exfoliate and expand when rapidly heated, due to the loss of water molecules between the silicate sheets. Studies by authors such as Midgley and Midgley [15] and Couderc and Douillet [16] have shown that the greatest exfoliation occurs in regular mica–vermiculite interstratified forms. Vermiculites with a higher potassium content in the interlayer have a lower water content and are less crystalline, with more interstratifications. Vermiculite is a low-cost mineral with abundant reserves, easy exfoliation, and high porosity and specific surface area [15,16,17].

Due to its unique physicochemical properties, in particular its thermal resistance and its ability to exfoliate, vermiculite has numerous industrial and technological applications. In the construction industry, it is appreciated for its insulating and lightweight characteristics, making it ideal for the production of concrete, plaster, rendering, fireproofing materials, and thermal pavements [18]. In addition, its particle size and intrinsic porosity make it suitable for a variety of technological uses, such as chemical process catalysts and fluid filtration media [19,20,21]. Recent research has also highlighted the potential of vermiculite in biomedical engineering, including cancer theranostics [22]. These diverse applications highlight the versatility of vermiculite, whose properties can be modified by physical, chemical, or mechanical treatments to meet the specific needs of different industries.

Hydrothermal treatment utilizes temperature and pressure to process materials like vermiculites [23,24]. These reactions can modify the chemical composition, crystalline structure, and texture of minerals, conferring distinctive physicochemical properties. These processes play a key role in natural environments, such as ore formation and geo-thermal systems, and are also crucial in industrial and technological applications like the production of synthetic materials [24]. Furthermore, this versatile method is also employed for waste management and resource recovery [25]. Hydrothermal techniques are used in materials science to modify the characteristics of materials and improve their functionality. Experimental factors such as fluid composition, temperature, and pressure are critical in determining the outcome of these reactions under hydrothermal conditions. Previous studies have analyzed several hydrothermal transformation processes related to the formation or alteration of vermiculite [23,24,26]. For instance, the employment of hydrothermal treatments has been proposed to increase vermiculite’s porosity, allowing the mineral to act as a molecular sieve for trapping CO_2_ molecules [27]. By controlling the experimental reaction conditions in hydrothermal treatments, the properties of commercial vermiculites can be tailored for several applications in adsorbents, catalysts, and nanomaterials production.

The mechanochemical process stands out for its low energy consumption, reduced processing temperatures, and cost-effectiveness. It offers a powerful, sustainable, time-efficient, environmentally friendly, and economical alternative for synthesizing functional materials. This process induces phase transformations in polymorphic solids, structural disorder, formation of solid solutions, ionic exchanges, complex formation, redox reactions, acid–base reactions, and polymer amorphization [28]. According to Baláž [29], the effects of mechanochemical processes are significant for various technological applications, including agriculture, extractive metallurgy, waste treatment, materials engineering, construction technology, pharmaceuticals, and the coal industry. Clark and Rowan [30] discovered that solid-state grinding or applying high pressure to certain compounds could produce similar structural effects. Grinding bentonite and kaolinite caused physical disintegration and particle size reduction and disruption of the crystalline structure [30,31]. Takahashi [32] and Garcia et al. [33] found that the dry mechanochemical treatment of pure kaolinite and natural clay resulted in disordered and amorphous crystal structures. The degree of amorphization during dry milling varied depending on the original mineral’s structural order.

In this study, hydrothermal and mechanochemical treatments were performed on three different Chinese commercial vermiculites. The objective was to produce synthetic materials with enhanced specific surface areas for industrial and technological applications. The responses of the investigated vermiculites to the applied treatments and their physicochemical properties were investigated by X-ray diffraction (XRD), and thermal analysis (TG and DTG), scanning electron microscopy (SEM); their textural characterization was analyzed by the BET method. Finally, the relationships between the physicochemical and structural properties of the investigated commercial vermiculites were analyzed.

## 2. Materials and Methods

### 2.1. Materials

**Figure 1:**
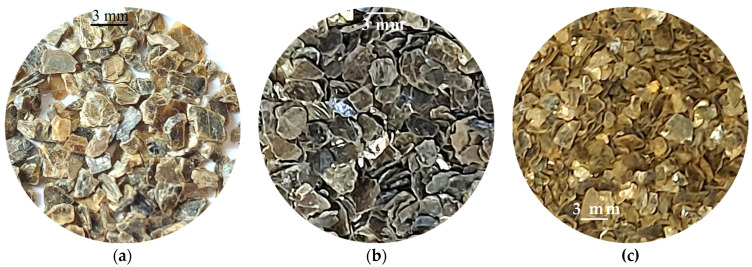
Appearance of the investigated vermiculites in hand samples: (**a**) CHG, (**b**) CHS, and (**c**) CHGO.

The vermiculites investigated in this paper come from China and they were supplied by China National Non-Metallic Minerals Industrial Import & Export Corporation (CNMIEC) (Beijing, China). The particle size of all the vermiculites was less than 5 mm in diameter, with thicknesses ranging from 0.5 to 1 mm. In appearance, the vermiculites had greenish, silver, and golden colors (Figure 1).

### 2.2. Experiments

Hydrothermal and mechanochemical experiments were made using the raw vermiculites without previous treatment, in order to preserve their natural characteristics as much as possible.

Hydrothermal experiments utilizing distilled water and CO_2_ were carried out in a steel reactor (BR-100, Berghof Products + Instruments GmbH, Eningen, Germany) equipped with a temperature–time controller (BTC-3000, Berghof Products + Instruments GmbH, Eningen, Germany), a pressure probe, and a gauge. The reactor contains a polytetrafluoroethylene (PTFE) vessel (total volume of 75 mL). For these experiments, raw vermiculite samples, of around 1.50 g, and 60 mL of distilled water were introduced into the PTFE vessel, which was subsequently positioned inside the steel reactor and sealed. The reactor was then connected to an industrial CO_2_ gas cylinder, and CO_2_ was allowed to flow for 3 min to expel the air. The valves were closed to achieve a CO_2_ pressure of 10 bars. The reactors were then heated to 100 °C and stirred at 200 rpm for a duration of 24 h. Following the experimental phase, the reactors were cooled to ambient temperature, and the pressure valves were opened to vent the residual CO_2_. The contents of the vessel were filtered through a 0.2 μm membrane filter (Millipore, Darmstadt, Germany), and the solid residue was left to dry at room temperature for 24 h. This methodology was reproduced using 60 mL acidic 0.1 M HCl solutions added to the raw vermiculites in the PTFE vessel at the beginning of the experiments (i.e., hydrothermal acid treatment).

The pH of the distilled water and the hydrothermal solutions at room temperature after separation of the solid phase was measured with a CRISON BASIC 20 pH meter, using a glass electrode (Metrohm, Filderstadt, Germany) calibrated with three standard solutions (pH = 4.01, 7.01 and 10.01 at 25 °C) with ±0.01 uncertainty.

Mechanochemical treatments were made using the raw vermiculites with the Retsch MM500 Vario (Sheffield, UK) equipment, with up to six grinds at a time. The frequency was set to 30 Hz for 30 min, using a 440C stainless steel Retsch screw closure jar of 10 mL with a thin PTFE seal, and one 10 mm diameter 440C stainless steel milling ball.

Both heating and microwave experiments were previously published [7,34].

The samples were labeled as shown in Table 1.

### 2.3. Sample Characterizations

The chemical composition of the initial samples was obtained by X-ray fluorescence from previously published research [35,36]. The major elements determined were the following: Al_2_O_3_, P_2_O_5_, K_2_O, CaO, SiO_2_, TiO_2_, MnO, Fe_2_O_3_, MgO, and Na_2_O.

The XRD patterns of the raw samples and those treated with the acid hydrothermal and mechanochemical methods, previously ground with an agate mortar, were taken with a PANalyticalX’pert Pro (Malvern Panalytical, Malvern, UK) diffractometer. Setting conditions were 40 mA and 45 kV (Cu-Kα radiation; λ = 1.5418 Å), 2θ range of 5–70 degrees (in which the most important phases are reflected), 2θ step scans of 0.007° and a counting time of 1 s per step. The standard reference material used was 660a NIST LaB6 with Full Width at Half Maximum (FWHM) of 0.06° for 2θ = 21.36°. The XRD patterns of the heated and microwave-irradiated samples were obtained from previously published research [35,36]. The software X’Pert HighScore Plus 2.2d (2.24) 2008 was used to identify the mineral composition, to detect changes in the intensity and position of the basal reflections, and to calculate the width at half height (FWHM) of the principal reflections; in addition, the crystallinity of the untreated and treated samples was also calculated.

The thermal gravimetric analyses were conducted between 25 and 1100 °C on the raw samples and those treated with the acid hydrothermal and mechanochemical methods. The equipment was a Mettler Toledo Stare System Thermobalance (Mettler Toledo, Columbus, OH, USA) with an alumina crucible, a heating rate of 10 °C/min, and flowing oxygen at 50 mL/min. The total mass loss was determined gravimetrically by heating the samples in air at 1000 °C.

The textural parameters of the raw powdered samples and the samples treated hydrothermally and mechanochemically were determined with the ASAP 2020 equipment (Iberfluid Instruments, Barcelona, Spain) under the following conditions: nitrogen adsorption at −195.8 K, with σ_m_ (N_2_) of 0.162 nm^2^; unrestricted evacuation of 30.0 mm Hg; vacuum pressure of 10 μm Hg; evacuation time of 1 h; and a temperature of the sample evacuation prior to N_2_ adsorption measurements of 22 °C. The data were recorded with equilibration times (P/P_0_ ranging between 0.001 and 1.000) between 50 and 25 s and a minimum equilibrium delay of 600 s at P/P_0_ ≥ 0.995. The specific surface area and pore size data have been determined by using a mathematical description of the adsorption isotherms with the software of the equipment. Three replicates were used in the experiments.

## 3. Results

The chemical analyses of the starting Chinese samples [35,36] showed a K_2_O percentage of 3.92 for CHG, 4.80 for CHS, and 5.08 for CHGO, high values indicating that these samples are composed of various interstratified layers of mica/vermiculite, vermiculite with different states of hydration, mixtures of mica and vermiculite, etc.; that is, the samples are “commercial vermiculites”, with the CHG sample being purer than the other two.

**Figure 2:**
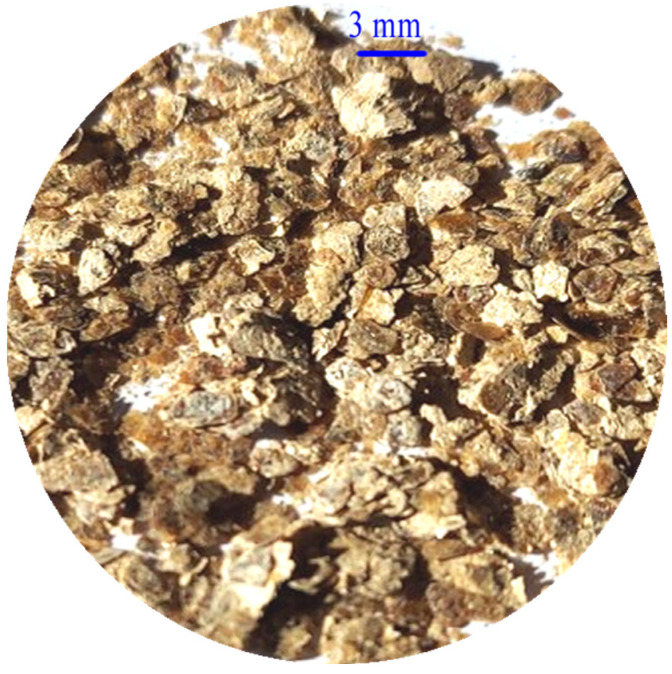
Syrupy appearance of the CHG sample after hydrothermal treatment.

**Figure 3:**
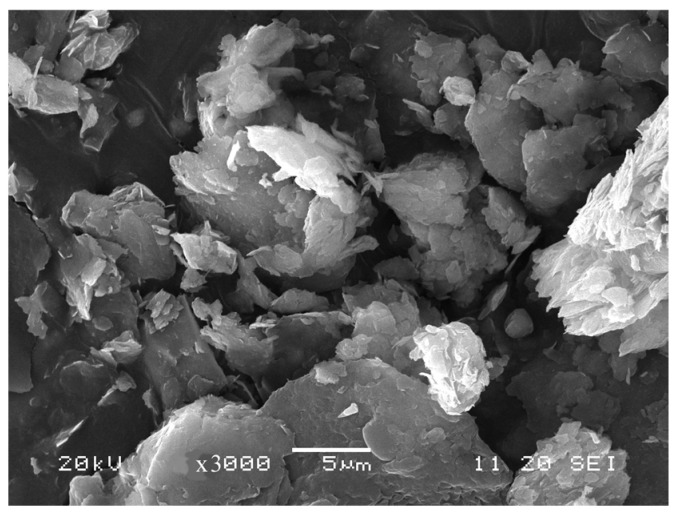
SEM image of the delaminated appearance of the CHGO sample after mechanochemical treatment.

After hydrothermal treatment, the Chinese vermiculites showed delamination and syrupy appearance (Figure 2, as example). The pH of the hydrothermal solutions after the filtration of the samples increased up to 1.5–2 compared with the pH of the acidic solutions (about pH = 1) used in the hydrothermal experiments. The mass loss in hydrothermally treated samples ranged between 1.7 and 3.2% except for the sample CHS-A which had a mass loss of 30%. The mechanochemically treated samples showed generally irregularly shaped and delaminated particles with sizes between 3 and 30 nm (CHG > CHGO > CHS) and of a gray color (Figure 3, as example).

**Figure 4:**
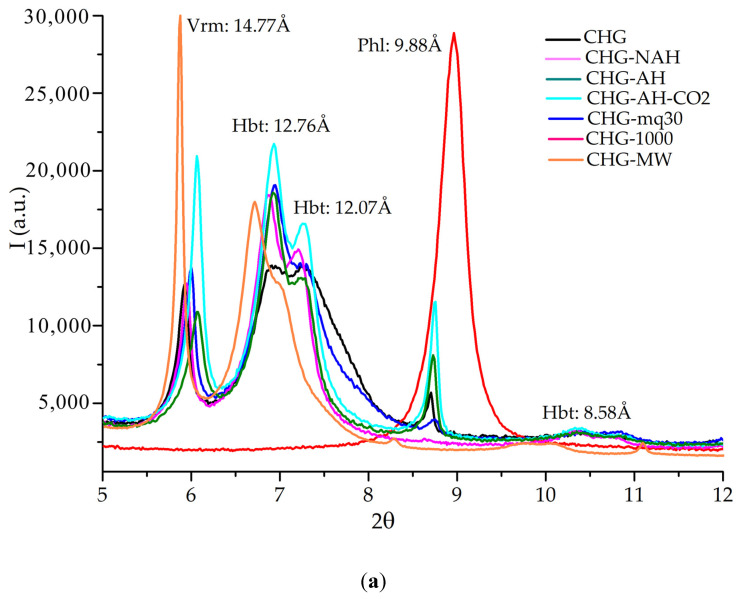

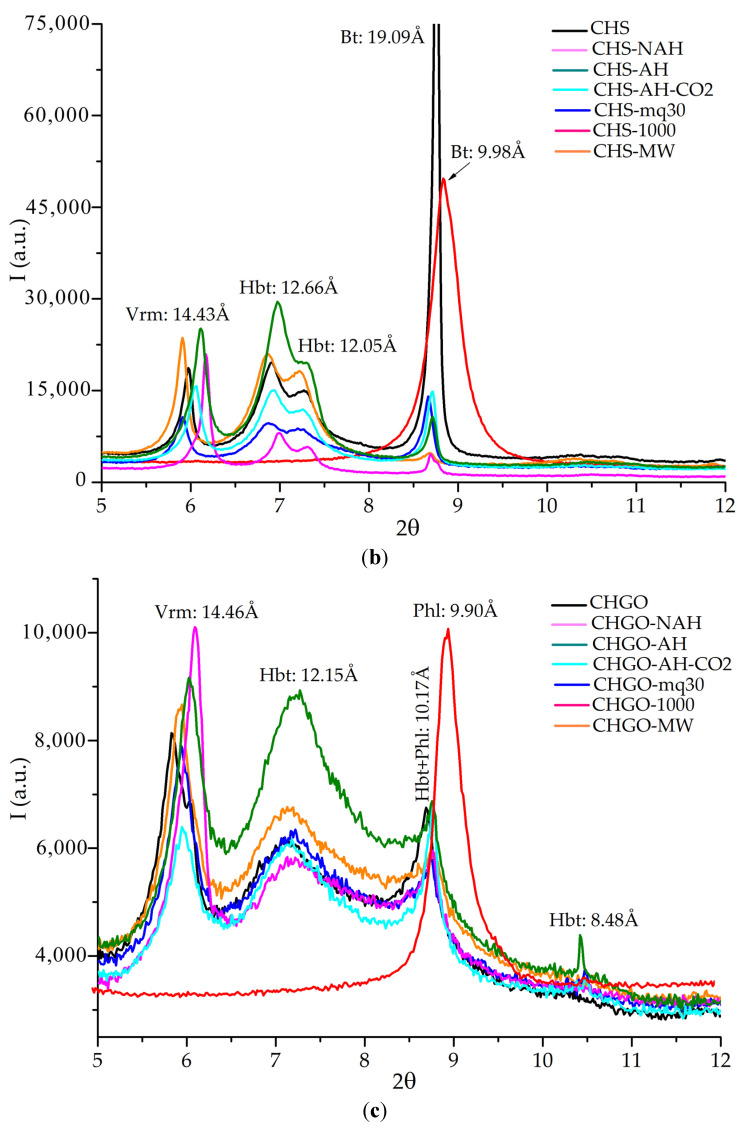
XRD of the untreated and treated samples of CHG (**a**), CHS (**b**), and CHGO (**c**). Note: Vrm = vermiculite, PHl = phlogopite, Bt = biotite, and Hbt = hydrobiotite.

The XRD spectra for the untreated and treated (at ambient T) samples of CHG, CHS, and CHGO are presented in Figure 4a–c, 5–12 °2θ range (Figure S1a–c, covering the 5–70 °2θ range); the principal reflections of the mineral phases of the investigated samples are highlighted. The mineral phases’ compositions are detailed in Table 2.

All the investigated samples, both untreated and treated, present a similar mineral composition (Figure 3 and Figure 4), varying in the percentage of the phases and their crystallinity and order, taking into account the intensity and width at half height (FWHM) of their main reflections (Table 3). In the CHG sample, the detected mineral phases were vermiculite, hydrobiotite, and a significantly smaller proportion of phlogopite. In the CHG-NAH sample, the intensity of hydrobiotite reflections increased, while phlogopite reflections decreased notably. In the CHG-AH sample, both vermiculite and phlogopite reflections intensified, but the vermiculite reflections decreased slightly, with the intensity of hydrobiotite reflections remaining unchanged. In CHG-AH-CO_2_, the intensity of reflections from vermiculite, hydrobiotite, and phlogopite increased, with vermiculite and hydrobiotite reflections showing similar intensities. In the CHG-mq30 sample, the intensity of hydrobiotite reflections was greater than that of vermiculite, which was stronger than that of phlogopite.

In the CHS sample, biotite, vermiculite, and hydrobiotite were detected, with biotite being the dominant phase. In CHS-NAH, the intensity of vermiculite reflections increased, while phlogopite reflections decreased. In CHS-AH, the intensity of hydrobiotite reflections increased. In CHS-AH-CO_2_, the intensity of hydrobiotite reflections increased, with a more significant increase for vermiculite. In CHS-MW, the intensity of vermiculite reflections increased.

In the CHGO sample, vermiculite, phlogopite, and hydrobiotite were the detected mineral phases. In CHGO-NAH, the intensity of vermiculite reflections increased, while phlogopite reflections decreased. In CHGO-AH, the intensities of both vermiculite and hydrobiotite reflections increased. In CHGO-AH-CO_2_, the intensity of vermiculite and phlogopite reflections decreased, especially for vermiculite. In CHGO-mq30, the intensities of hydrobiotite and phlogopite reflections decreased.

In the CHG samples, the crystallinity value varies between 24.7% for CHG-NAH and 36.2 for CHG. In the CHS samples, the crystallinity value varies between 28.2% for CHS-mq30 and 58.4 for CHS. In the CHGO samples, the crystallinity value varies between 12.3% for CHGO-AH-CO_2_ and 18.8 for CHGO-1000. The CHS sample has the highest crystallinity values and CHGO has the lowest values.

**Figure 5:**
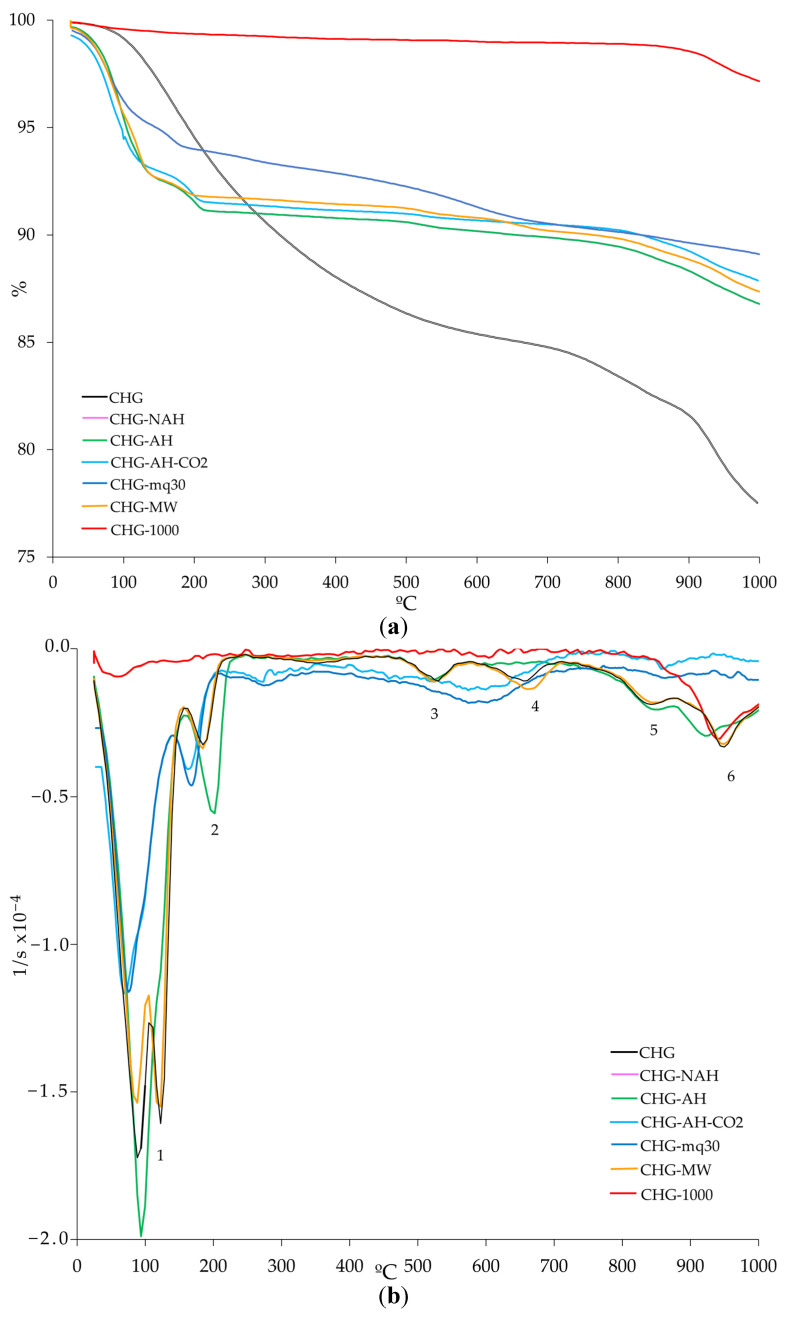

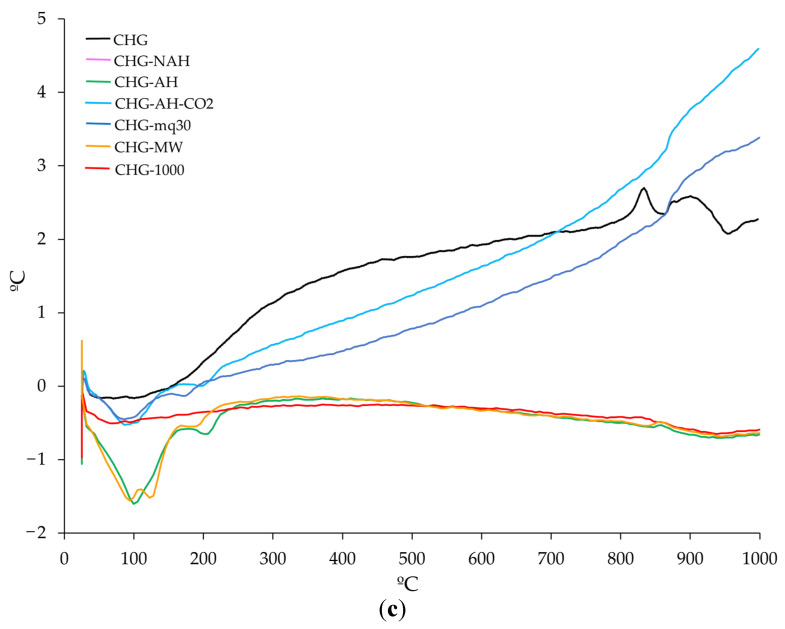
Curves of TG (**a**) and DTG (**b**) and SDTA (**c**) for the untreated and treated samples of CHG (heating rate of 10 °C/min and flowing oxygen at 50 mL/min). Note: 1—adsorbed surface water loss; 2—interlayer water loss and interlayer cation-bound water; 3–4—loss of hydroxyls; 5—decomposition of CO_2_; 6—recrystallization.

**Figure 6:**
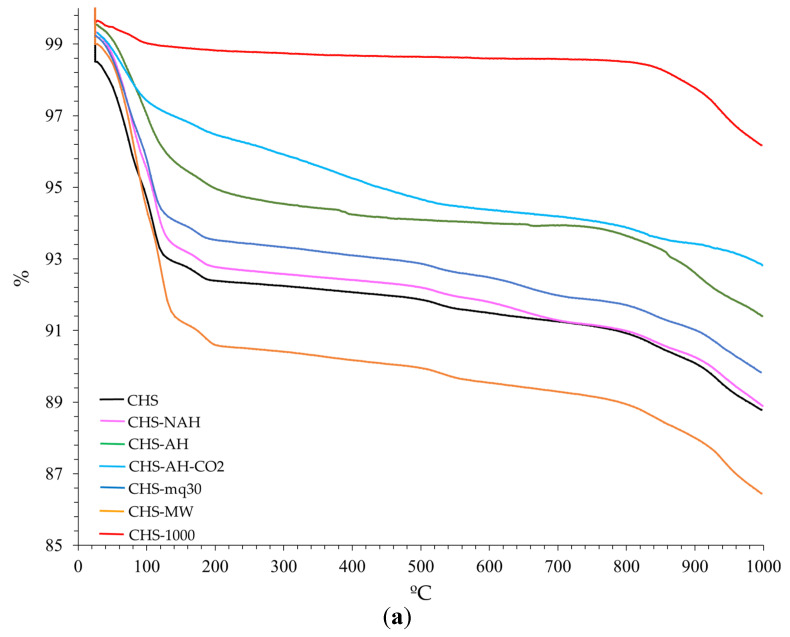

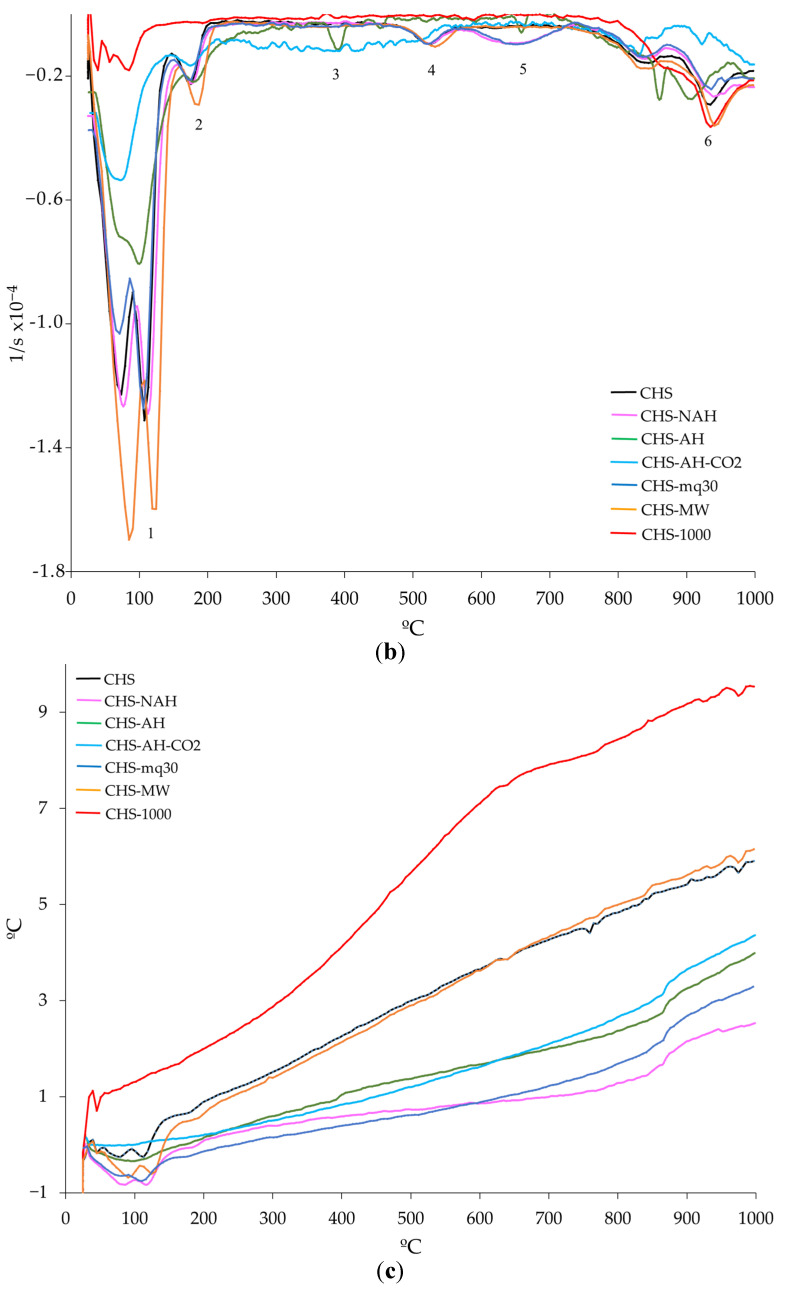
Curves of TG (**a**) and DTG (**b**) and SDTA (**c**) for the untreated and treated samples of CHS (heating rate of 10 °C/min and flowing oxygen at 50 mL/min). Note: 1—adsorbed surface water loss; 2—interlayer water loss and interlayer cation-bound water; 3–4—loss of hydroxyls; 5—decomposition of CO_2_; 6—recrystallization.

**Figure 7:**
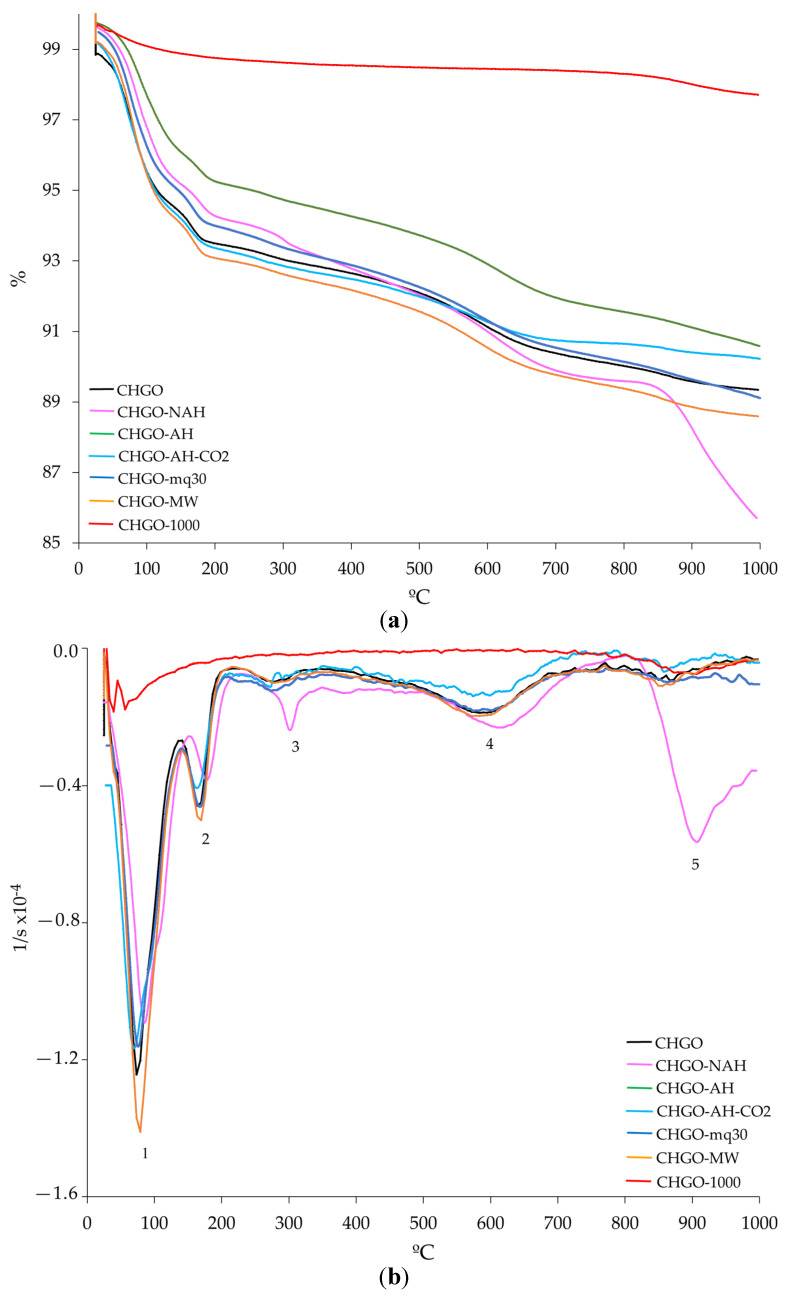

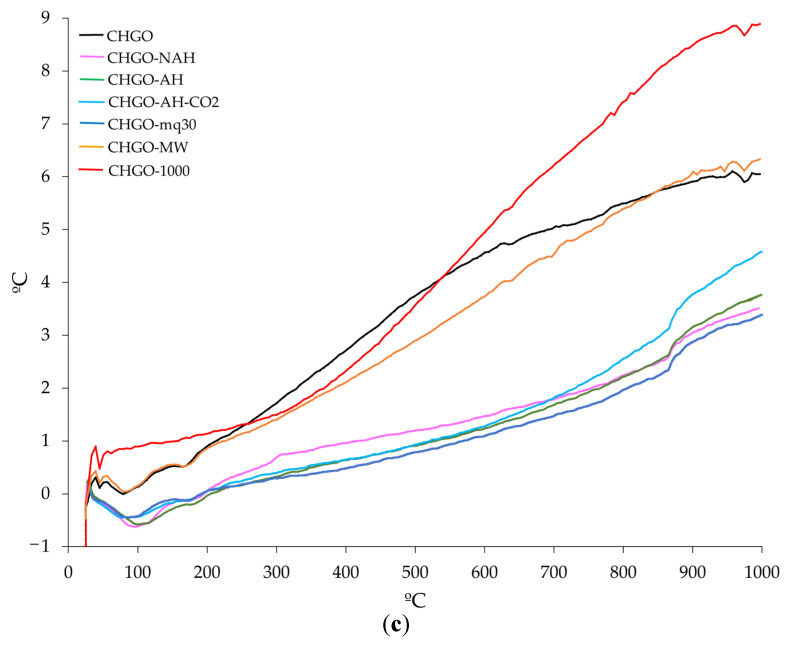
Curves of TG (**a**) and DTG (**b**) and SDTA (**c**) for the untreated and treated samples of CHGO (heating rate of 10 °C/min and flowing oxygen at 50 mL/min). Note: 1—adsorbed surface water loss; 2—interlayer water loss and interlayer cation-bound water; 3—loss of hydroxyls; 4—decomposition of CO_2_; 5—recrystallization.

The TG, DTG, and SDTA curves of the starting and treated samples of CHG, CHS, and CHGO are presented in Figure 5, Figure 6, and Figure 7, respectively. The TG curves of all investigated samples (Figure 5a, Figure 6a and Figure 7a, respectively) show two steps. The first step, from 25 to about 250 °C, is due to the loss of adsorbed water on the surface and/or localized in the interlayer space of the vermiculites [37]. The second step, at temperatures above 800 °C, shows an additional mass loss due to recrystallization into new phases or to a transformation process associated with the dehydroxylation of OH^−^ anions in the octahedral layer [7,37]. From the TG curves, the mass loss was calculated (Table 4). In general, all the treated samples lost less mass than the untreated ones. Water loss varied significantly among the treated samples, with CHS losing the least and CHG the most.

Up to five different steps can be observed in the DTG curves of the untreated and treated samples of each type of vermiculite analyzed (Figure 5b, Figure 6b, and Figure 7b, respectively) The first step, ranging from approximately 50 to 150 °C, is due to the loss of surface adsorbed water. In a second step, at temperatures above 200 °C, a loss of the interlayer water and the water bound to the interlayer cations is observed. The third step, at temperatures between 550 °C and 650 °C, is due to the loss of hydroxyls. The fourth step is due to CO_2_ decomposition, and the fifth step, at temperatures above 850 °C, is due to phase recrystallization.

The SDTA curve of each analyzed sample (Figure 5c, Figure 6c, and Figure 7c, respectively) indicates endothermic processes; from 850 °C, the baseline of the SDTA curve becomes exothermic, probably due to a phase change in the solid state.

**Figure 8:**
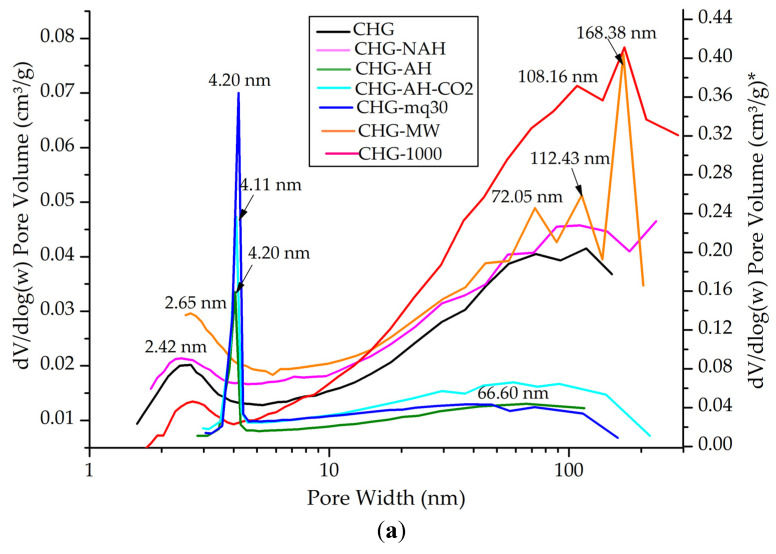

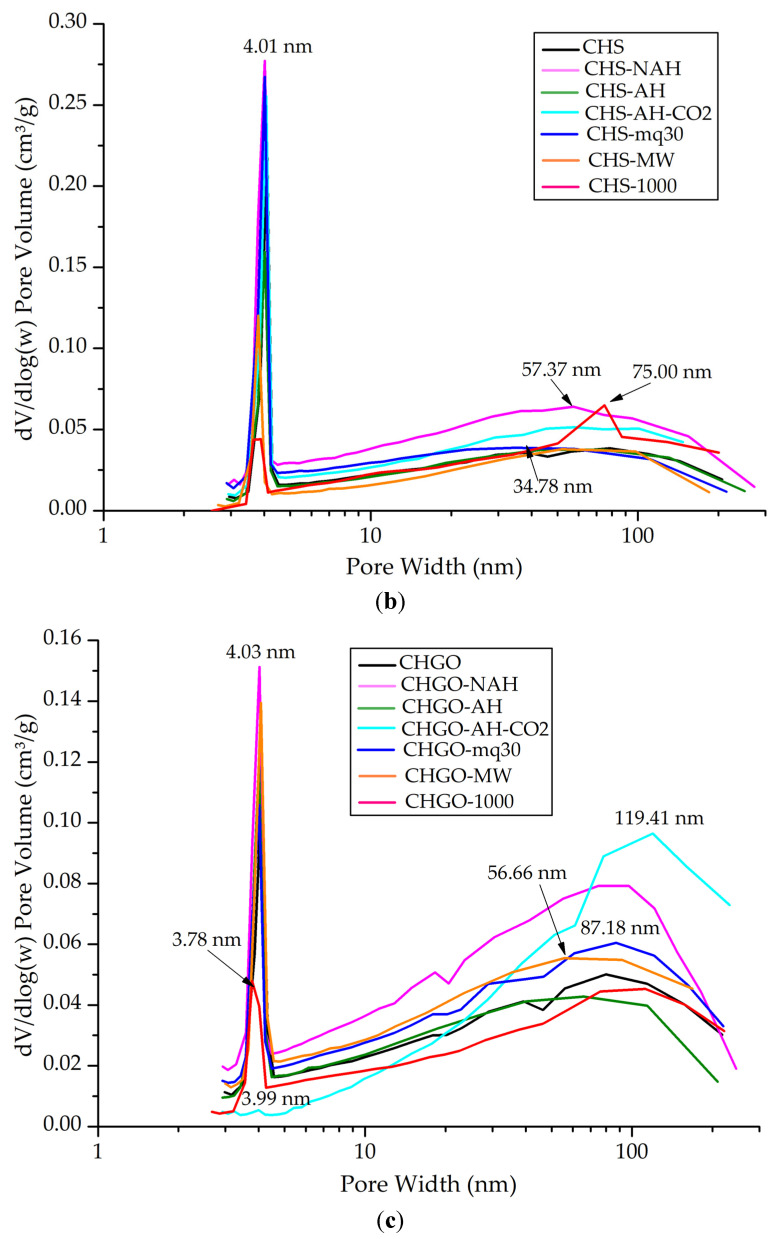
Pore size vs. dV/dlog(w) graph: (**a**) CHG samples (* for CHG-AH, CHG-AH-CO_2_, and CHG-mq30), (**b**) CHS samples, (**c**) CHGO samples.

The nitrogen adsorption–desorption isotherms of the investigated vermiculites (Figure S2a–c) correspond to type IV and V of the IUPAC classification, with characteristics of mesoporous solids [38]. This type of isotherm exhibits a hysteresis loop, H3 type, which is associated with capillary condensation taking place in mesopores, and it has a limited uptake over a range of high P/P_0_; this type is characteristic of layered particles, such as the phyllosilicate group minerals, including vermiculite [15]. The hysteresis for both the untreated and the treated samples, at a relative pressure (P/P_0_) of about 0.5, is similar in the CHG and CHS samples, but slightly more pronounced in CHGO. The peaks in the dV/dlog(w) pore volume (cm^3^/g) vs. pore width (nm) indicate the most predominant pore sizes in the material (Figure 8a–c). The specific surface area (S_BET_), adsorption capacity (Q_m_), average cumulative pore volume (V_p_) and the corresponding pore width (nm), BET constant (C), and correlation coefficient (R^2^) values obtained from the adsorption–desorption experiments are shown in Table 5. The specific surface area (S_BET_) was measured using the BET mathematical model. The model used for the pore size calculation has been the Barrett–Joyner–Halenda (BJH) model [39], which is applied only to type IV isotherms, considering the Faass correction [40], which adjusts for the change in thickness of the multilayers during the intervals in which the cores are not emptied.

The highest S_BET_ and Q_m_ values were shown by the hydrothermally CO_2_-treated samples, CHGO-NAH and CHS-NAH, indicating a higher surface area and adsorption capacity (27.7–31.7 m^2^/g and 0.28–0.32 mmol/g), followed by the mechanochemically treated sample CHG-mq30. Vermiculites heated to 1000 °C abruptly for 1 min provided the lowest S_BET_ and Q_m_ values. The S_BET_ and crystallinity correlations for the CHG samples were moderately positive (0.396), suggesting that higher crystallinity might be associated with a slight increase in surface area. For the CHS samples, the correlation was very weakly negative (−0.033), and for the CHGO samples, it was very weakly positive (0.044), indicating in both cases that changes in crystallinity do not significantly affect the surface area. The CHG samples demonstrate a more significant relationship between crystallinity and S_BET_, whereas the CHS and CHGO samples show that changes in crystallinity do not substantially influence the surface area.

In the CHG sample, two distinct pore size trends were observed: mesopores, ranging from 2.40 nm (CHG-AH, CHG-AH-CO_2_, and CHG-mq30) to 2.60 nm (CHG, CHG-NAH, CHG-MW, and CHG-1000); and macropores, with sizes between approximately 70 nm and 170 nm, which occupy more volume. In contrast, the CHS and CHGO samples are characterized by mesopores with sizes between 3.80 nm and 4.00 nm, which occupy more volume, and macropores, ranging from 34.78 nm to 75.00 nm in the CHS samples and from 56.66 nm to 119.41 nm in the CHGO samples. For the CHG samples, the largest average pore size was observed in CHG-MW (282.46 nm), and CHG-AH had the smallest (14.91 nm). Among the CHS samples, CHS had the largest average pore size (26.40 nm), whereas CHS-MW had the smallest (19.53 nm). Among the CHGO samples, CHGO-AH had the largest average pore size (210.95 nm), whereas CHGO-MW had the smallest (16.77 nm).

In the CHG samples, the correlation between crystallinity and pore size was moderately negative (−0.368). In the CHS samples, the correlation was very weakly negative (−0.033). In the CHGO samples, the correlation was also very weakly negative (−0.233). The relationship between crystallinity and pore size varied significantly across the sample sets: in the CHG samples, higher crystallinity tended to reduce the pore size; in the CHS samples, it tended to increase the pore size; and in the CHGO samples, it slightly reduced the pore size.

The C values of the investigated untreated and treated samples, ranging between 67 and 198, confirmed the validity of the BET method. The C values that were higher, up to 150, were generally associated either with adsorption on high-energy surface sites or the filling of narrow micropores. The C values that were lower than 50 indicate there is an appreciable overlap of monolayer and multilayer adsorption [38].

The R^2^ values above 0.999 indicated the high correlation of the data and a good model fit.

**Figure 9:**
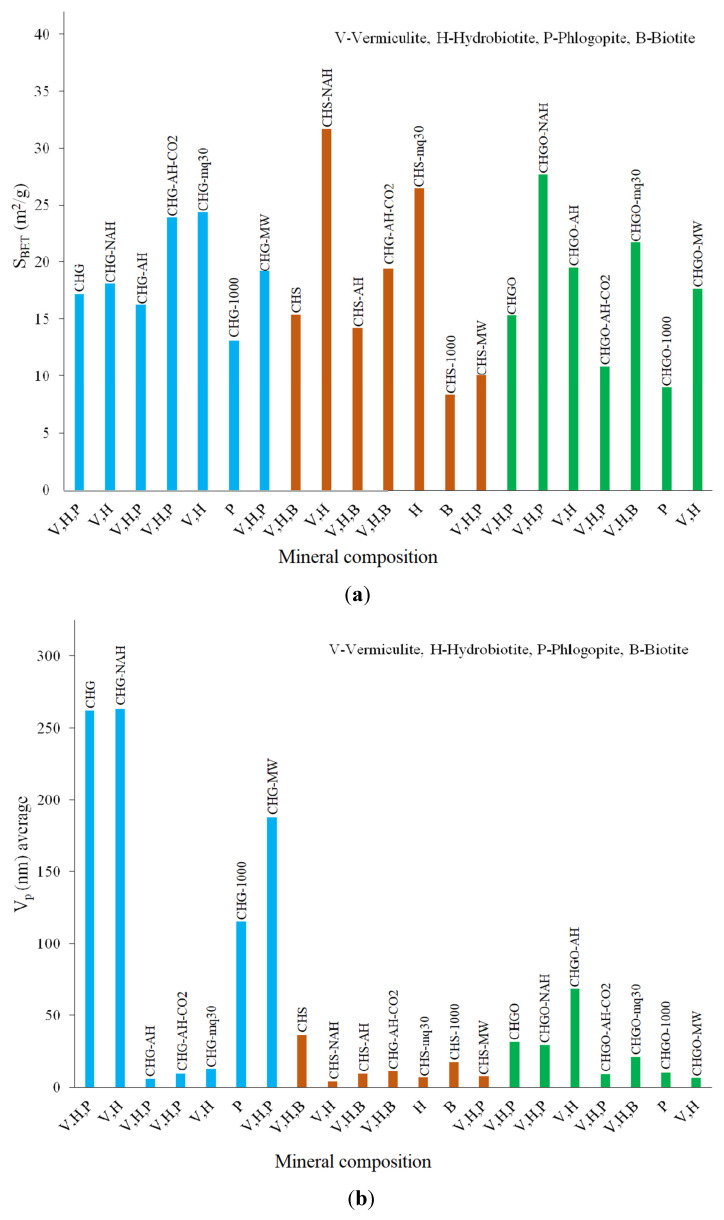

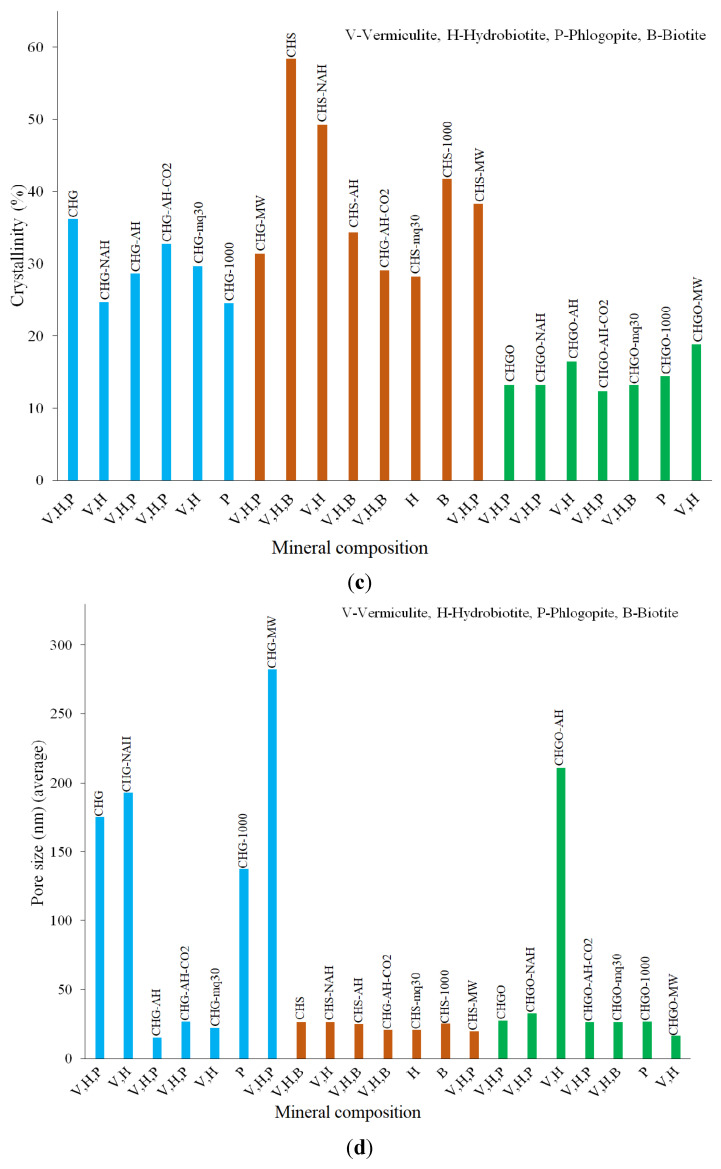
Relationships between mineral composition and S_BET_ (**a**), mineral composition and average V_p_ (**b**), and mineral composition and crystallinity (**c**); and the relationship between crystallinity and average pore size (**d**).

The relationships between mineral composition and S_BET_, mineral composition and average V_p_, mineral composition and crystallinity, and crystallinity and average pore size are shown in Figure 9a–d. The relationship between mineral composition and S_BET_ shows that the samples with vermiculite, hydrobiotite, and phlogopite generally have moderate to high S_BET_ values. The samples with vermiculite and hydrobiotite show variability, with mechanochemical treatments significantly increasing S_BET_. The samples with phlogopite, especially those treated at high temperatures, tend to have lower S_BET_ values. The samples with biotite also show variability, with some treatments significantly reducing S_BET_. The relationship between mineral composition and average V_p_ shows that the samples with vermiculite, hydrobiotite, and phlogopite generally have moderate V_p_ values. The samples with vermiculite and hydrobiotite show variability, with mechanochemical treatments significantly increasing V_p_. The samples with phlogopite, especially those treated at high temperatures, tend to have lower V_p_ values. The samples with biotite also show variability, with some treatments significantly reducing V_p_. The most effective treatments for increasing V_p_ are the hydrothermal, mechanochemical, and hydrothermal CO_2_ treatments.

## 4. Discussion

The pH variation in hydrothermal solutions (between 1 and 2) is influenced by the dissolution of CO_2_ in water, which releases protons and lowers the pH, making the solution more acidic. The slight pH increase observed at the end of the experiment suggests that, after the initial vermiculite dissolution under acidic conditions, OH^−^ groups from the vermiculite structure are released into the solution. This is likely due to the replacement of CO_2_ by structural water and OH^−^ groups, similar to previous observations of CO_2_ or alcohol incorporation into vermiculite [27,34,41].

The mass loss in the hydrothermally treated samples is attributed to the leaching of vermiculite elements like sodium, silicon, magnesium, potassium, and calcium, as well as to water loss [27]. Consequently, the pressure decreased during hydrothermal treatment in all cases, with a more uniform decrease in samples CHS and CHGO (around 4 bars) compared to CHG (6–8 bars).

All investigated samples, both untreated and treated, exhibit similar mineral compositions. However, they vary in phase percentages, crystallinity, and order, as indicated by the intensity and width at half height of their main reflections. Comparing these samples with those abruptly heat-treated at 1000 °C for 1 min [35,36] reveals the following transformations: in the CHG sample, vermiculite and hydrobiotite transformed into phlogopite, with the main reflection intensity increasing by approximately 400%; in the CHS sample, vermiculite and hydrobiotite transformed into biotite, with the main reflection intensity increasing by approximately 56%; in the CHGO sample, vermiculite and hydrobiotite transformed into phlogopite, with the main reflection intensity increasing by approximately 550%. When comparing the samples in this study with those subjected to microwave irradiation [35,36], we observe the following: the CHG and CHGO samples showed reflection intensities similar to the raw samples; in the CHS sample, the intensity of vermiculite reflections increased.

The loss of crystallinity and structural order in the vermiculites studied is due to water loss from various treatments (hydrothermal in acidic and non-acidic aqueous solutions with and without CO_2_, mechanochemical). The significant variation in crystallinity across samples indicates that the different treatments applied affect their crystalline structure, which in turn influences their material properties, such as mechanical strength and thermal stability. Higher crystallinity generally improves mechanical strength and thermal stability.

In general, all treated samples lost less mass than untreated ones. Water loss varied significantly among treated samples, with CHS losing the least and CHG the most. With hydrothermal treatment, water is lost through CO_2_ substitution leading to vermiculitization. With mechanochemical treatment, dehydration is produced by stress, and in the samples CHG and CHGO, a vermiculitization is produced, and the CHS sample is transformed to hydrobiotite. The vermiculites heated at 1000 °C abruptly for 1 min lost very little water (0.5–0.8%) because their water content was much lower after the heat pre-treatment. The microwave irradiation treatment of the investigated vermiculites resulted in a much lower water loss than when subjected to sudden high temperature heating.

Crystallinity affects S_BET_, average V_p_, and average pore size. The crystallinity and S_BET_ correlations that are moderately positive suggest that higher crystallinity might be associated with a slight increase in surface area. The very weak negative or positive correlations could indicate that crystallinity changes do not significantly affect the surface area. These correlations would indicate that the effect of crystallinity on S_BET_ can vary significantly depending on the mineralogical composition of the sample and the specific treatments applied. Higher crystallinity can reduce S_BET_ due to a more ordered structure and fewer defects. It also influences pore size and distribution, resulting in fewer or smaller pores, which impacts adsorption capacity and molecule diffusion. The trends between crystallinity and pore size in the analyzed samples highlight the importance of mineral composition and structural interactions. The specific mineral phases and their ratios in each sample set play a crucial role in tailoring material properties.

In relation to the practical implications of the changes of S_BET_ and V_P_, we expose the significance of these differences by focusing on the treated vermiculite samples, particularly comparing CHG-mq30 and CHG-AH-CO_2_. The CHG-mq30 sample has a high specific surface area, similar to that of CHG-AH-CO_2_ (23.9 m^2^/g) and a significantly larger pore volume (12.87 cm^3^/g) compared to CHG-AH-CO_2_ (9.50 cm^3^/g). A larger pore volume indicates more space within the material for storing or transporting molecules. This makes CHG-mq30 particularly suitable for applications requiring high storage capacity, such as in gas storage, drug delivery systems, or as a support material in catalysis. CHG-AH-CO_2_ has a smaller pore volume, which suggests a denser structure with less internal space. While it still has a high surface area, the smaller pore volume may limit its capacity for applications requiring large storage or transport volumes. However, it could be advantageous in applications where a more compact structure is needed, such as in certain types of filtration or as a structural component in composites. Understanding these practical implications can help in selecting the appropriate treatment method and material for specific industrial and technological applications.

The relationship between crystallinity and average pore size shows an inverse trend; higher crystallinity tends to create smaller average pore sizes due to fewer defects and larger pores. However, significant variability in the data suggests other factors may also influence pore size. The pore size data demonstrate that specific treatments such as hydrothermal treatment on the samples investigated can effectively increase pore size, for applications requiring high surface area and permeability. The analysis of the graph dV/dlog(w) pore volume (cm^3^/g) vs. pore width (nm) confirmed that the general trend is decreasing with significant peaks at certain pore widths. These peaks indicate that there are certain pore widths where the material has a higher volume capacity, which may be relevant for specific applications requiring materials with high storage capacity in certain pore sizes.

Higher crystallinity generally improves mechanical strength and thermal stability but can reduce specific surface area (S_BET_) and pore size due to fewer defects and a more compact structure. Lower crystallinity often means more defects in the crystal structure, such as vacancies, dislocations, and interstitial atoms. These defects can create additional surface area and increase pore volume. Increased structural disorder can lead to the formation of amorphous regions within the material, which typically have higher surface areas and larger pore sizes compared to crystalline regions. The formation of hydrobiotite, a mixed-layer mineral, can occur during treatments like hydrothermal processing. This phase transformation can disrupt the regular structure of vermiculite, leading to increased porosity and surface area. Phase transformations can create new pore structures and increase the overall pore volume. For example, the transformation from vermiculite to hydrobiotite can introduce additional interlayer spaces, contributing to larger pore sizes.

The crystallinity of CHG drops from 36.2% (untreated) to 24.7% (CHG-NAH). The specific surface area increases from 17.2 m^2^/g to 18.1 m^2^/g. The reduction in crystallinity likely introduces more defects and structural disorder, increasing the surface area. The hydrothermal treatment may also promote the formation of hydrobiotite, which can further enhance porosity. The CHG-AH-CO_2_ sample shows a similar S_BET_ (23.9 m^2^/g) but with a smaller pore volume (Vp = 9.50 cm^3^/g) compared to CHG-mq30. The acidic conditions and CO_2_ presence can lead to significant phase transformations, such as the formation of hydrobiotite, which can increase surface area but result in a denser structure with smaller pores. Higher crystallinity materials, like untreated CHG, offer better mechanical strength and thermal stability, making them suitable for structural applications.

The inverse relationship between crystallinity and pore size is influenced by the introduction of defects, structural disorder, and phase transformations. Lower crystallinity increases surface area and pore volume by creating more defects and amorphous regions. Phase transformations, such as hydrobiotite formation, further contribute to these changes by disrupting the regular structure and enhancing porosity. Understanding these mechanisms helps in tailoring vermiculite properties for specific applications.

The relationship between mineral composition and crystallinity shows that the samples with vermiculite, hydrobiotite, and phlogopite tend to have moderate to high crystallinity values, ranging from approximately (15–25%). The samples with vermiculite and hydrobiotite show variability in crystallinity (10–30%). The samples with phlogopite samples, especially those treated at high temperatures, tend to have lower crystallinity values (below 15%). In conclusion, the samples with biotite also show variability in crystallinity (8–20%). The samples with vermiculite and hydrobiotite generally have higher crystallinity than those with mainly phlogopite or biotite.

## 5. Conclusions

The mineral composition of both treated and untreated samples (100–1000 °C) is similar, though the treatments modify the intensity and width of phase reflections, altering crystallinity and structural order, as well as the proportions of vermiculite, hydrobiotite, and phlogopite. The treated samples generally exhibit lower mass loss, influencing crystallinity.

The isotherms reveal mesoporous features, with the hydrothermally CO_2_-treated samples showing the highest surface area and adsorption capacity.

Mechanochemical treatments significantly enhance S_BET_ and pore volume (V_p_) in vermiculite and hydrobiotite. Heat treatments promote the transformation of vermiculite and hydrobiotite into phlogopite or biotite, increasing crystallinity and improving mechanical and thermal properties.

Controlling crystallinity is key, as higher crystallinity can reduce S_BET_ due to a more ordered structure and fewer defects, but it enhances mechanical strength and thermal stability. Crystallinity directly affects pore size, distribution, and adsorption capacity, with an inverse correlation between crystallinity and pore size. This relationship enables the design of materials with specific pore structures.

These findings highlight the influence of various treatments on the structural, chemical, and textural properties of vermiculite, offering valuable guidance for developing materials tailored to industrial and technological applications by enabling precise control over these properties after such processing.

## Figures and Tables

**Table 1:** Labels of the starting and treated vermiculite samples for each experiment conducted.

Treatments						
Starting	Hydrothermal (No Acid)	Hydrothermal (Acid)		Mechanochemical (30 min)	Heating (1000 °C)	Irradiation (Microwave)
		Without CO_2_	With CO_2_			
CHG	CHG-NAH	CHG-AH	CHG-AH-CO_2_	CHG-mq30	CHG-1000	CHG-MW
CHS	CHS-NAH	CHS-AH	CHS-AH-CO_2_	CHS-mq30	CHS-1000	CHS-MW
CHGO	CHGO-NAH	CHGO-AH	CHGO-AH-CO_2_	CHGO-mq30	CHGO-1000	CHGO-MW

**Table 2:** The mineral phase compositions of the investigated samples. Note 1: ^a^ Published by Marcos et al. [35], ^b^ published by Lahchich et al. [36]. Note 2: JCPDs = Joint Committee on Powder Diffraction Standards.

Sample	Mineral Phase Composition
CHG	Vermiculite (JCPDs 16-613), Phlogopite (JCPDs 11-295), Hydrobiotite (JCPDs 10-363)
CHG-NAH ^a^	Vermiculite (JCPDs 10-418), Phlogopite (JCPDs 10-495), Hydrobiotite (JCPDs 2-38)
CHG-AH	Vermiculite (JCPDs 34-166), Phlogopite (JCPDs 10-495), Hydrobiotite (JCPDs 10-358)
CHG-AH-CO_2_	Vermiculite (JCPDs 10-418), Phlogopite (JCPDs 10-495), Hydrobiotite (JCPDs 10-363)
CHG-mq30	Vermiculite (JCPDs 2-21), Hydrobiotite (JCPDs 10-362)
CHG-MW ^a^	Vermiculite (JCPDs 16-613), Phlogopite (JCPDs 10-495), Hydrobiotite (JCPDs 13-233)
CHG-1000 ^a^	Phlogopite (JCPDs 16-344)
CHS	Vermiculite (JCPDs 34-166), Biotite (JCPDs 46-1440), Hydrobiotite (JCPDs 10-358)
CHS-NAH	Vermiculite (JCPDs 34-166), Biotite (JCPDs 42-603), Hydrobiotite (JCPDs 13-233)
CHS-AH	Vermiculite (JCPDs 34-166), Biotite (JCPDs 42-603), Hydrobiotite (JCPDs 13-233)
CHS-AH-CO_2_	Vermiculite (JCPDs 34-166), Biotite (JCPDs 42-603), Hydrobiotite (JCPDs 13-233)
CHS-mq30	Vermiculite (JCPDs 2-21), Hydrobiotite (JCPDs 13-233)
CHS-MW	Vermiculite (JCPDs 34-166), Phlogopite (JCPDs 10-492), Biotite (JCPDs 46-1440), Hydrobiotite (JCPDs 13-233)
CHS-1000 ^b^	Biotite (JCPDs 42-1437)
CHGO	Vermiculite (JCPDs 34-166), Phlogopite (JCPDs 10-492), Hydrobiotite (JCPDs 10-358)
CHGO-NAH	Vermiculite (JCPDs 2-21), Phlogopite (JCPDs 10-495), Hydrobiotite (JCPDs 10-363)
CHGO-AH	Vermiculite (JCPDs 16-613), Phlogopite (JCPDs 10-495), Hydrobiotite (JCPDs 13-466)
CHGO-AH-CO_2_	Vermiculite (JCPDs 16-613), Phlogopite (JCPDs 16-344), Hydrobiotite (JCPDs 13-233)
CHGO-mq30	Vermiculite (JCPDs 2-21), Hydrobiotite (JCPDs 10-362), Biotite (JCPDs 42-1437), Quartz (JCPDs 5-490), Magnesium Sulfate Oxide (JCPDs 1-549)
CHGO-MW	Vermiculite (JCPDs 34-166), Phlogopite (JCPDs 10-495), Hydrobiotite (JCPDs 10-363)
CHGO-1000 ^b^	Phlogopite (JCPDs 2-53)

**Table 3:** The FWHM (2θ) and crystallinity (%) values of the main reflections of the phases present in the untreated and treated samples of CHG, CHS, and CHGO.

Sample	FWHM (2θ)				Crystallinity (%)
	Vermiculite	Hydrobiotite	Phlogopite	Biotite	
CHG	0.1023	0.2303	0.0512		36.2
CHG-NAH	0.0768	0.1535			24.7
CHG-AH	0.1023	0.1407	0.0768		28.6
CHG-AH-CO_2_	0.0895	0.1535	0.0640		32.7
CHG-mq30	0.0895	0.1919			29.6
CHG-MW	0.1023	0.0640	0.1279		24.5
CHG-1000			0.2175		31.4
CHS	0.0768	0.1535		0.0512	58.4
CHS-NAH	0.0640	0.0895			49.2
CHS-AH	0.1023	0.1663		0.0768	34.3
CHS-AH-CO_2_	0.1023	0.1407		0.0768	29.1
CHS-mq30		0.0895			28.2
CHS-MW	0.0768	0.1535	0.0768		41.7
CHS-1000				0.6140	38.3
CHGO	0.0768	0.307	0.0768		13.2
CHGO-NAH	0.1407	0.5118	0.0640		13.2
CHGO-AH	0.1663	0.3582			16.4
CHGO-AH-CO_2_	0.2303	0.1791	0.0768		12.3
CHGO-mq30	0.0512	0.4605		0.0640	13.2
CHGO-MW	0.0512	0.5628	0.1535		14.4
CHGO-1000			0.6140		18.8

**Table 4:** Loss of mass (%) of the investigated samples. Note: ^a^ Published by Marcos et al. [35], ^b^ published by Lahchich et al. [36].

	Total Mass Loss (%)	Hydration Water Loss (%)
CHG	22.4	7.0
CHG-NAH ^a^	13.3	9.0
CHG-AH	11.7	8.2
CHG-AH-CO_2_	11.4	7.8
CHG-mq30	12.4	8.0
CHG-MW ^a^	4.2	7.9
CHG-1000 ^a^	2.2	0.5
CHS	15.5	6.8
CHS-NAH	5.2	1.7
CHS-AH	8.2	4.7
CHS-AH-CO_2_	6.5	2.9
CHS-mq30	9.4	5.2
CHS-MW	3.2	8.5
CHS-1000 ^b^	2.4	0.8
CHGO	11.5	8.1
CHGO-NAH	14.0	5.5
CHGO-AH	9.2	4.6
CHGO-AH-CO_2_	9.0	6.0
CHGO-mq30	10.0	5.4
CHGO-MW	3.8	6.2
CHGO-1000 ^b^	2.4	0.8

**Table 5:** Specific surface area (S_BET_), adsorption capacity (Q_m_), average pore volume (V_p_) and the corresponding pore width, BET constant (C), and correlation coefficient (R^2^) of nitrogen adsorption–desorption measurements for raw and treated vermiculites. Note: ^a^ Published by Marcos et al. [35], ^b^ published by Lahchich et al. [36], except for average pore volume (V_p_) and average pore width.

Sample	S_BET_ (m^2^/g)	Q_m_ (mmol/g)	V_p_ (cm^3^)	Pore Width (nm)	C	R^2^
CHG	17.2 ± 0.1	0.18	261.91	174.97	93	0.9999
CHG-NAH ^a^	18.1 ± 0.2	0.19	262.97	192.94	70	0.9992
CHG-AH	16.2 ± 0.1	0.16	5.86	14.91	116	0.9999
CHG-AH-CO_2_	23.9 ± 0.1	0.24	9.50	26.77	120	0.9998
CHG-mq30	24.4 ± 0.1	0.25	12.87	22.05	193	0.9999
CHG-MW ^a^	19.2 ± 0.2	0.20	187.72	282.46	80	0.9995
CHG-1000 ^a^	13.1 ± 0.1	0.13	115.00	137.41	76	0.9999
CHS	15.4 ± 0.1	0.16	36.35	26.40	138	0.9998
CHS-NAH	31.7 ± 0.1	0.32	4.03	26.26	115	0.9999
CHS-AH	14.2 ± 0.1	0.14	9.60	24.96	98	0.9998
CHS-AH-CO_2_	19.4 ± 0.1	0.20	11.16	20.74	105	0.9998
CHS-mq30	26.5 ± 0.1	0.27	6.78	20.66	198	0.9998
CHS-MW	10.0 ± 0.1	0.10	7.39	19.53	67	0.9996
CHS-1000 ^b^	8.33 ± 0.1	0.09	17.50	25.22	86	0.9997
CHGO	15.3 ± 0.1	0.16	31.63	27.15	100	0.9997
CHGO-NAH	27.7 ± 0.1	0.28	29.44	32.71	129	0.9999
CHGO-AH	19.5 ± 0.1	0.20	68.12	210.95	110	0.9998
CHGO-AH-CO_2_	10.8 ± 0.0	0.11	9.37	26.42	126	0.9999
CHGO-mq30	21.7 ± 0.1	0.22	20.82	26.36	145	0.9998
CHGO-MW	17.6 ± 0.2	0.18	6.45	16.77	191	0.9996
CHGO-1000 ^b^	9.0 ± 0.1	0.09	10.12	26.75	135	0.9998

## Data Availability

The original contribution presented in the study are included in the article, and further inquiries can be directed to the corresponding author.

## Supplementary Materials

The following supporting information can be downloaded at: https://www.mdpi.com/article/10.3390/ma18040831/s1, Figure S1: XRD of untreated and treated samples of CHG (a), CHS (b) and CHGO (c) in the range of 2 theta 5–70°. Note: Vrm = vermiculite, PHl = phlogopite, Bt = biotite, and Hbt = hydrobiotite. The phases indicated correspond to their position on the untreated sample.; Figure S2: The nitrogen adsorption-desorption isotherms of the untreated and treated samples CHG (a), CHS (b) and CHGO (c).
