# Supplementary material for: Relationships Between Physicochemical and Structural Properties of Commercial Vermiculites
Source: Materials (Basel). 2025 Feb 14;18(4):831. doi: 10.3390/ma18040831 (PMC11857825; doi:10.3390/ma18040831)
Supplement: Supplementary file 1 [file materials-18-00831-s001.zip › materials-3443333-supplementary.pdf]

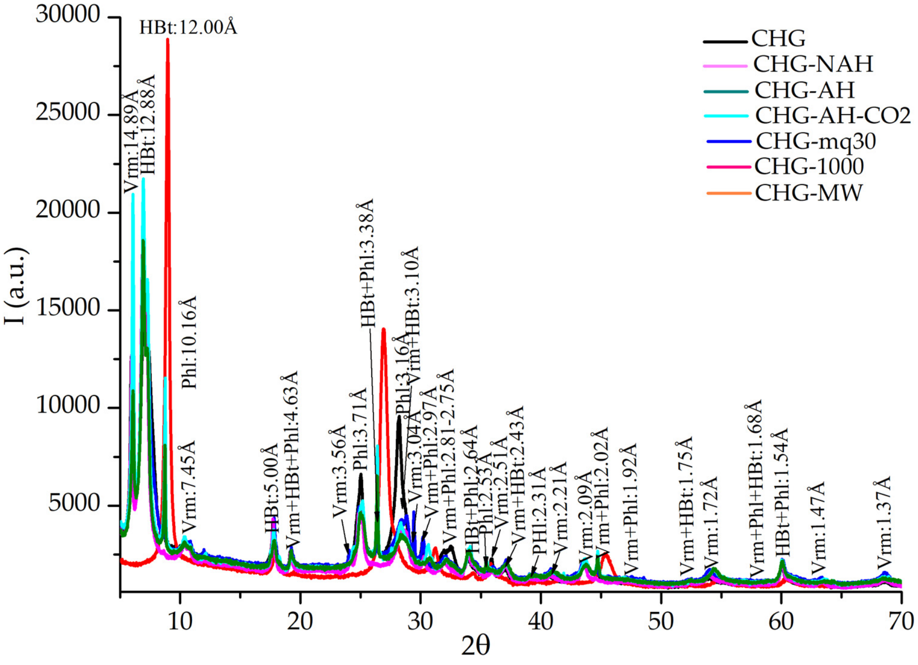
CHG
CHG-NAH
CHG-AH
CHG-AH-CO2
CHG-mq30
CHG-1000
CHG-MW
HBt:12.00Å
Vrm:14.89Å
HBt:12.88Å
Phl:10.16Å
Vrm:7.45Å
HBt:5.00Å
Vrm+HBt+Phl:4.63Å
Vrm:3.56Å
Phl:3.71Å
HBt+Phl:3.38Å
Vrm+HBt:3.10Å
Phl:3.16Å
Vrm:3.04Å
Vrm+Phl:2.97Å
Vrm+Phl:2.81-2.75Å
HBt+Phl:2.64Å
Phl:2.53Å
Vrm:2.51Å
Vrm+HBt:2.43Å
PHl:2.31Å
Vrm:2.21Å
Vrm:2.09Å
Vrm+Phl:2.02Å
Vrm+Phl:1.92Å
Vrm+HBt:1.75Å
Vrm:1.72Å
Vrm+Phl+HBt:1.68Å
HBt+Phl:1.54Å
Vrm:1.47Å
Vrm:1.37Å
I (a.u.)
2θ
(a)

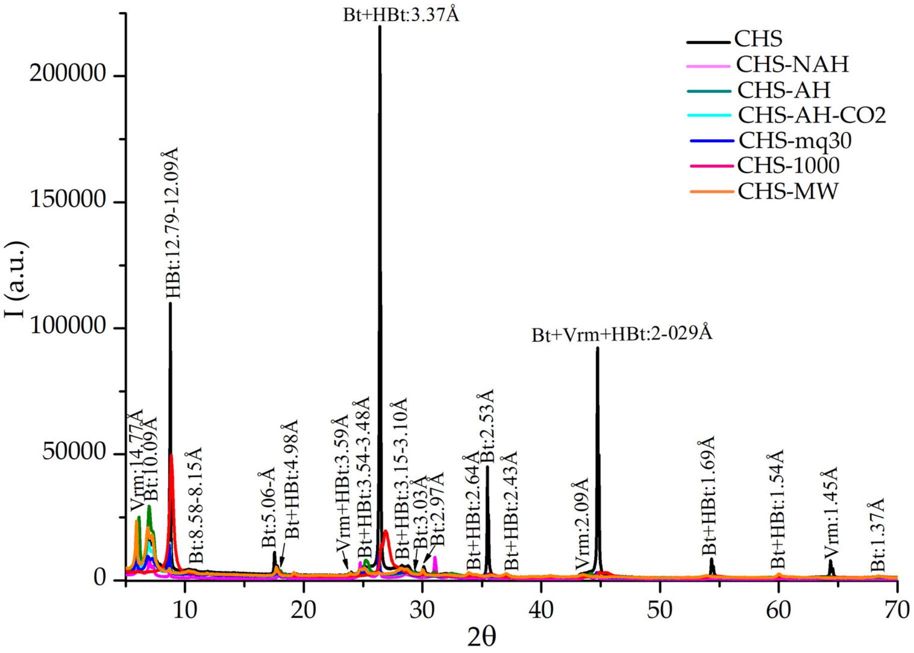
CHS
CHS-NAH
CHS-AH
CHS-AH-CO2
CHS-mq30
CHS-1000
CHS-MW
Bt+HBt:3.37Å
HBt:12.79-12.09Å
Bt+Vrm+HBt:2-029Å
Vrm:14.77Å
Bt:10.09Å
Bt:8.58-8.15Å
Bt:5.06-Å
Bt+HBt:4.98Å
Vrm+HBt:3.59Å
Bt+HBt:3.54-3.48Å
Bt+HBt:3.15-3.10Å
Bt:3.03Å
Bt:2.97Å
Bt+HBt:2.64Å
Bt:2.53Å
Bt+HBt:2.43Å
Vrm:2.09Å
Bt+HBt:1.69Å
Bt+HBt:1.54Å
Vrm:1.45Å
Bt:1.37Å
I (a.u.)
2θ
(b)

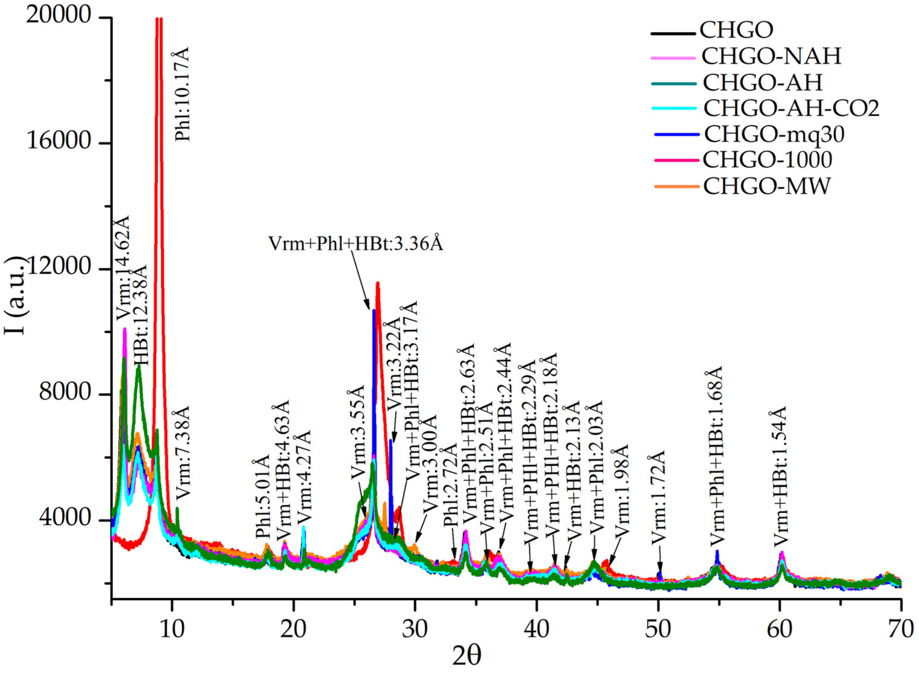

(c)

Figure S1. XRD of untreated and treated samples of CHG (a), CHS (b) and CHGO (c) in the range of 2 theta 5-70 º. Note: Vrm = vermiculite, PHl = phlogopite, Bt = biotite, and Hbt = hydrobiotite. The phases indicated correspond to their position on the untreated sample.

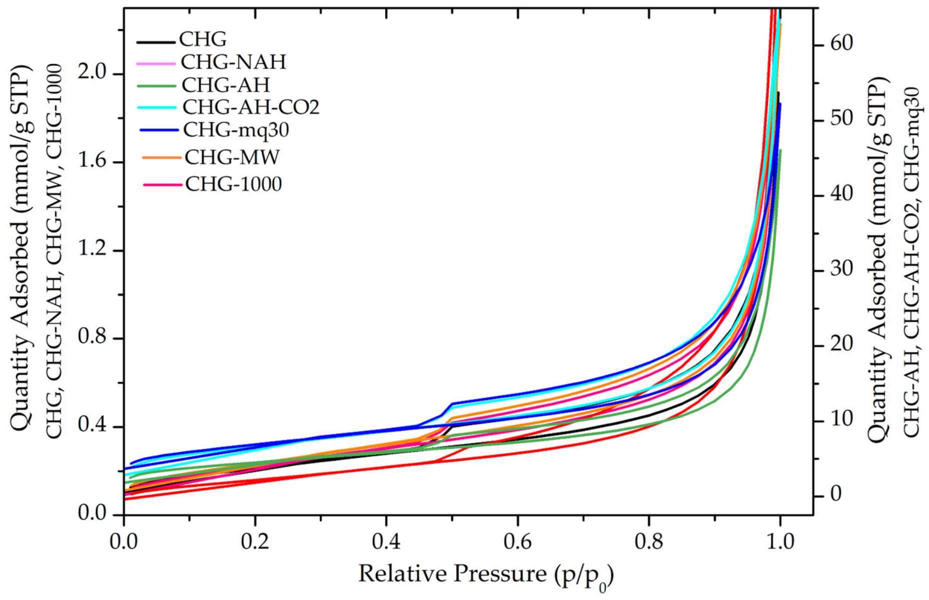

(a)

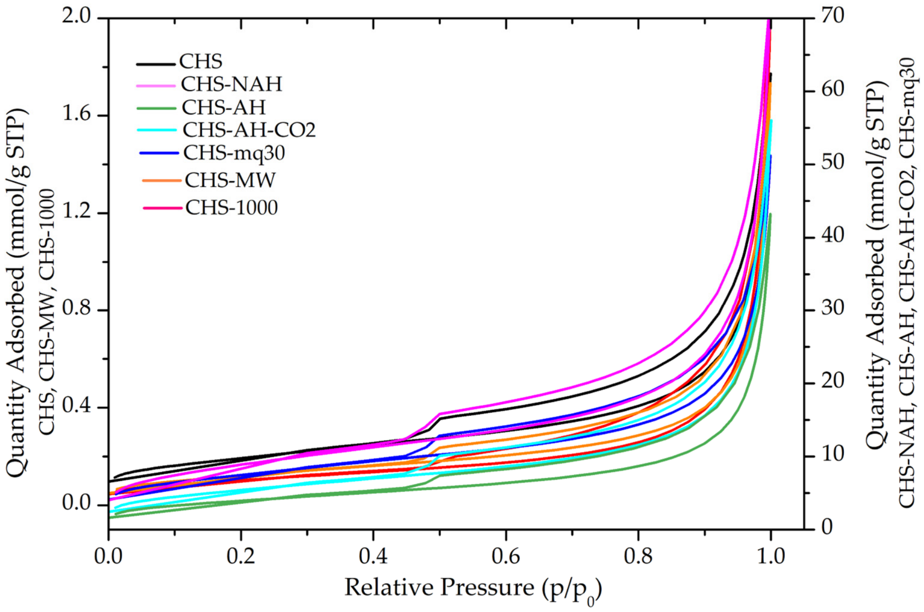

(b)

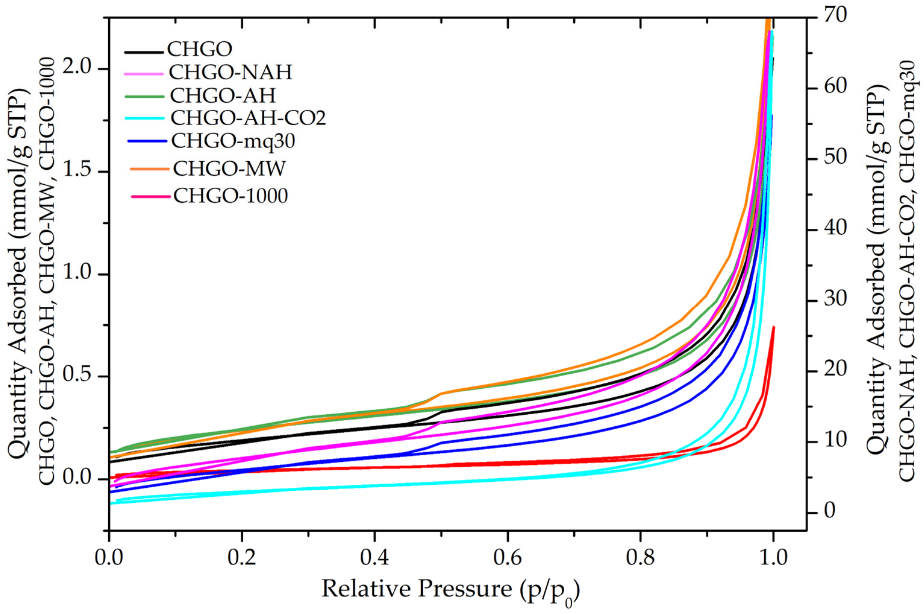

(c)

Figure S2. The nitrogen adsorption-desorption isotherms of the untreated and treated samples CHG (a), CHS (b) and CHGO (c).
